# PHOX2A and PHOX2B are differentially regulated during retinoic acid-driven differentiation of SK-N-BE(2)C neuroblastoma cell line

**DOI:** 10.1016/j.yexcr.2016.02.014

**Published:** 2016-03-01

**Authors:** Simona Di Lascio, Elena Saba, Debora Belperio, Andrea Raimondi, Helen Lucchetti, Diego Fornasari, Roberta Benfante

**Affiliations:** a Department of Medical Biotechnology and Translational Medicine (BIOMETRA), Università degli Studi di Milano, Milan, Italy; b CNR – Neuroscience Institute, Milan, Italy; c San Raffaele Scientific Institute, Imaging Research Centre, Milan, Italy

**Keywords:** α3 nAChR, alpha 3 nicotinic Acetylcholine Receptor, ATRA, *all-trans* Retinoic Acid, BMP-2, Bone morphogenetic protein-2, CCHS, Congenital Central Hypoventilation Syndrome, Cdk, cyclin-dependent kinase, ChIP, chromatin immunoprecipitation, CKI, Cdk inhibitor, DβH, dopamine-β-hydroxylase, DR, directed repeat, GDNF, glial derived neurotrophic factor, HSCR, Hirschprung's disease, NB, neuroblastoma, NT3, neurotrophin 3, RAR, retinoic acid receptor, RARE, retinoic acid responsive element, RXR, retinoid X receptor, TH, tyrosine hydroxylase, TH-MYCN, tyrosine hydroxylase‑*v*‑myc avian myelocytomatosis viral oncogene, Neuroblastoma, Retinoic acid, Human, Transcription, Transcription factor, Differentiation, Homeodomain protein

## Abstract

PHOX2B and its paralogue gene PHOX2A are two homeodomain proteins in the network regulating the development of autonomic ganglia that have been associated with the pathogenesis of neuroblastoma (NB), because of their over-expression in different NB cell lines and tumour samples. We used the SK-N-BE(2)C cell line to show that all-*trans* retinoic acid (ATRA), a drug that is widely used to inhibit growth and induce differentiation in NBs, regulates both PHOX2A and PHOX2B expression, albeit by means of different mechanisms: it up-regulates PHOX2A and down-regulates PHOX2B. Both mechanisms act at transcriptional level, but prolonged ATRA treatment selectively degrades the PHOX2A protein, whereas the corresponding mRNA remains up-regulated. Further, we show that PHOX2A is capable of modulating PHOX2B expression, but this mechanism is not involved in the PHOX2B down-regulation induced by retinoic acid. Our findings demonstrate that PHOX2A expression is finely controlled during retinoic acid differentiation and this, together with PHOX2B down-regulation, reinforces the idea that they may be useful biomarkers for NB staging, prognosis and treatment decision making.

## Highlights

• RA regulates both PHOX2A and PHOX2B expression by means of different mechanisms.
• PHOX2A and PHOX2B expression must be controlled during RA induced differentiation.
• PHOX2A modulates PHOX2B, but does not mediate the RA-induced PHOX2B down-regulation.
• RA induces PHOX2A protein degradation by means of the ubiquitin-proteasome system.

## 1 Introduction

A neuroblastoma, one of the most frequent tumours of childhood, is caused by the arrested differentiation of neural crest sympatho-adrenal progenitor cells [1]. PHOX2B, and its paralogue PHOX2A, are two homeodomain transcription factors that play a pivotal role in the development of the autonomic nervous system and specification of the neurotransmitter phenotype by controlling the expression of the two enzymes responsible for noradrenaline biosynthesis (tyrosine hydroxylase [*TH*] and dopamine-β-hydroxylase [*DβH*]), and thus directing neurons towards their terminal noradrenergic differentiation [2], [3]. PHOX2B also modulates its own expression by means of an auto-regulatory mechanism [4] and the expression of *PHOX2A* [5], [6], whereas PHOX2A regulates the expression of the human *α3* nAChR subunit gene [7]. Both therefore play a primary role in controlling a number of the molecular determinants of autonomic neurons.

PHOX2A and PHOX2B are also involved in coordinating cell cycle exit and the differentiation of neural progenitors during sympathetic neuronal differentiation [8] as a result of their ability to induce the transcription of p27^Kip1^ [9], [10], [11], a cyclin-dependent kinase inhibitor (CKI) whose expression is also regulated by retinoic acid (RA) at post-translational level [12], followed by PHOX2B down-regulation during final neuronal differentiation [13].

Recently, the *PHOX2A* gene has been localised to near the deletion breakpoint of a number of 11q-deleted NB specimens [14], and microarray expression analysis has shown that it is one of nine noradrenaline biosynthesis pathway genes whose expression is reduced in unfavourable NB tumours [14]. However, the possible contribution of PHOX2A to the pathogenesis of NB is not univocal as it is over-expressed in a number of NB tumours and cell lines [15]. As no mutations have been observed in the *PHOX2A* regulatory or coding regions of tumour samples [14], [16], it is likely that this gene is involved in the pathogenesis of NB when its expression is deregulated in either direction.

The pathogenetic role of PHOX2B in NB is supported by the presence of heterozygous mutations in familial, sporadic and syndromic cases of NB, and its over-expression in tumour samples and NB cell lines, sometimes associated with other neurochristopathies such as Congenital Central Hypoventilation Syndrome (CCHS) and Hirschprung's disease (HSCR) [16], [17], [18], [19], [20], [21], [22], [23], but the underlying mechanisms are still largely unknown. *In vitro* and *in vivo* studies have linked the PHOX2B mutations associated with NB with the impaired differentiation of immature sympathetic neurons that can proliferate, and aberrant differentiation towards the glial lineage [10], [24]. PHOX2B over-expression leads to contradictory results as some studies indicate that it inhibits the proliferation of motoneuron progenitors and of immature sympathetic neurons [8], [10], [16] and promotes the differentiation of human NB cells after treatment with RA [16], whereas conditional Phox2b knockout studies have revealed that Phox2b is required for the proliferation of immature sympathetic neurons [25], and Alam et al. [13], and Ke et al. [23] have shown that a high level of PHOX2B promotes neuroblastoma cell proliferation and xenograft tumour growth in the TH-MYCN murine model, and that this correlates with a high level of MYCN expression. Furthermore, the presence of aberrant Phox2b expression in a zebrafish model has shown that the correct amount of the *Phox2b* gene is important for the differentiation of sympathetic neurons [26].

Vitamin A (retinol) profoundly affects various biological processes during development and adulthood. Most of its actions are mediated by its metabolic product, retinoic acid, which binds to specific nuclear receptors: heterodimers of retinoic acid receptors (RARs) *α*, *β* and *γ*, and retinoid X receptors (RXRs) *α*, *β* and *γ*. These ligand-activated receptors regulate gene transcription by binding to retinoic acid responsive elements (RAREs) in the promoter regions of responsive genes [27]. At embryological level, retinoids control the proliferation, migration and differentiation of neural crest-derived progenitors and, in developing sympathetic neurons, RA cooperates with Bone morphogenetic protein-2 (BMP-2) to make cells responsive to neurotrophic factors such as glial derived neurotrophic factor (GDNF) and neurotrophin 3 (NT3) [28], [29]. The pleiotropic effects of RA on the regulatory network governing sympathetic neuron differentiation are well known, but very little is known about its effect on the transcription factors (such as MASH1, PHOX2A and PHOX2B) that play a fundamental role in this process. *In vitro*, retinoids arrest cell growth in the G1 phase of the cell cycle, and induce differentiation in human NB cell lines [30], [31] along neuronal- or glial-like lineages depending on the cell line [32] by regulating, for example, the expression of *p27*^*Kip1*^, a target gene of PHOX2A and PHOX2B that has major functions in controlling the cell cycle.

As the target genes mediating retinoid-induced differentiation are largely unknown, and the molecular mechanisms by which RA regulates the different signalling pathways necessary for retinoid-induced cellular differentiation in various tissues and at different times are poorly understood, we tested the hypothesis that there may be a direct regulatory link between RA and PHOX2A and PHOX2B expression/activity in the SK-N-BE(2)C NB cell line. The findings show that the retinoic-acid induced differentiation of SK-N-BE(2)C cells is accompanied by a differential regulation of *PHOX2A* and *PHOX2B* expression, with up-regulation of *PHOX2A* mRNA followed by the disappearance of PHOX2A protein (the mRNA remains stably expressed), and a marked decrease in the expression of *PHOX2B* mRNA and protein, thus suggesting that their expression must be finely controlled during RA-induced differentiation, reinforcing the idea that they may be useful biomarkers for NB staging, prognosis and treatment decision making.

## 2 Material and methods

### 2.1 Cell lines and cultures

The SK-N-BE(2)C and IMR32 human neuroblastoma cell lines were grown in RPMI 1640, 10% fetal calf serum, 100 units/ml penicillin, 100 μg/ml streptomycin, and 2 mM l-glutamine (Lonza). All-*trans* retinoic acid (ATRA; Sigma-Aldrich, St.Louis, Missouri, USA), dissolved in 100% EtOH, was added at a final concentration of 10 μM for the times described in the and the medium was changed every day. Each treatment was carried out in duplicate and repeated at least three times in independent experiments using different batches of ATRA. Cycloheximide (Sigma-Aldrich, St. Louis, MO, USA) was added at a final concentration of 10 μg/ml before or after ATRA for the times described in the The proteasome inhibitor MG-132 (8 μM; Calbiochem, Darmstadt, Germany) was added for eight and 24 h after initial treatment with ATRA for 24 and 48 h.

### 2.2 Total RNA extraction, reverse transcription, and quantitative real-time PCR

Total RNA was extracted and reverse transcribed, and gene expression was quantitatively analysed as described by Benfante et al. [33] with minimal modifications. The TaqMan^®^ primer and probe assays (Life Technologies, Inc., Carlsbad, CA, USA) were *PHOX2A* (ID #Hs00605931_mH) and *PHOX2B* (ID #Hs00243679_m1), and glyceraldehyde-3-phosphate dehydrogenase (*GADPH*; ID# Hs99999905_m1) was used as an endogenous controls after its compatible with the other assays had been confirmed. The results were calculated using the 2^−ΔCT^ and the 2^−ΔΔCT^ methods in order to allow the normalisation of each sample to the endogenous control, and comparison with the calibrator of each experiment (set to a value of 1) as described in the figure legends.

### 2.3 Nuclear run-on

Nuclear run-on transcription was performed in accordance with the protocol described by Patrone et al. [34]. The nuclei (5×10^7^) were prepared from SK-N-BE(2)C cells treated for 24 h with ATRA or vehicle. RNA was synthesised *in vitro* by adding an equal volume of transcription buffer containing 0.4 mM biotin-16-UTP (Roche Diagnostics SpA, Monza, Milan, Italy), and the biotin-labelled RNA was isolated by means of streptavidin-coated magnetic beads (Dynabeads M-280 Streptavidin, Dynal Biotech ASA, Oslo, Norway). Gene expression was quantitatively analysed by reverse transcribing 8 µl of the nuclear RNA sample and 1 µg of the total RNA sample and using real-time PCR.

### 2.4 Chromatin immunoprecipitation and qPCR

Chromatin immunoprecipitation was carried out as previously described [4]. Chromatin was incubated overnight at 4 °C with 5 µg of anti-PHOX2A antibody (Davids Biotechnologie, Regensburg, Germany), and chicken pre-immune IgY (Davids Biotechnologie), and the immunocomplexes were collected on monoclonal anti-chicken IgY-agarose beads or protein G/agarose bead slurry (InVitrogen, Carlsbad, CA, USA) pre-adsorbed with 20 µg/µl tRNA and 10 µg/µl salmon sperm DNA (Sigma-Aldrich). After washes and elution, the cross-linking was reversed by heating to 65 °C overnight, and the samples were purified on columns (High Pure PCR product purification kit, Roche Diagnostics SpA, Italy). For the PCR detection of the immunoprecipitated chromatin, 5% of the purified DNA was used as a template to amplify the PHOX2B promoter using the primers ChIP[2 bprom] UP, 5′-CAA GCT TAT TTC CAA GTA GTG TGA TTG AAT-3′, and ChIP[2bprom] LOW, 5′-GCC TCC TAT GAG ATG CCT TGT CTG A-3′. The DNA samples were heated to 95 °C for 2 min, followed by 47 cycles of heating at 95 °C for 30 s, annealing at 64 °C for 30 s, and extension at 72 °C for 30 s. For quantitative analysis, the immunoprecipitated chromatin was amplified by means of SYBR-Green chemistry (Life Technologies, Inc.) using the primers #UP, 5′-GCT CGG TGC GTA ATG GTG TGG TA-3′ and #LOW, 5′-GGT TGG TCT TAT TGC TGG CGC TT-3′, and quantitatively analysed using the ABI Prism™ 7000 Sequence Detection System (Applied Biosystems, CA, USA) and SDS software, version 1.2.3.

### 2.5 shRNA

*PHOX2A* expression was silenced by means of the transient transfection of a plasmid (MISSION® shRNA, Cod. SHCLNG-NM_005169, TRCN0000013543, **Clone ID:**NM_005169.2-1260s1c1) containing an shRNA targeting the 3′-UTR region of *PHOX2A* (*Sequence:* CCG GCC TTC TAG CTT GGC CTT CTT TCT CGA GAA AGA AGG CCA AGC TAG AAG GTT TTT) and the results were analysed as described in the SHC002 MISSION® pLKO.1-puro Non-Mammalian shRNA Control Plasmid DNA (Sigma-Aldrich) was used as the control.

### 2.6 Electrophoretic mobility shift assays (EMSAs)

The EMSAs were performed as described by Terzano et al. [35] and Cargnin et al. [4]. The oligonucleotides spanning the ATTA sites in the *PHOX2B* promoter have been described by Cargnin et al. [4] and all of the oligonucleotides were purchased from Sigma Aldrich.

### 2.7 Plasmid construction

The β-RARE luc construct was obtained by cloning the consensus RARE from the β2 RA receptor subunit promoter [36] upstream of the SV40 promoter (Promega Madison, WI, USA). The oligonucleotide sequence was 5′-TCG AGT AAG GGT TCA CCG AAA GTT CAC TCG CAC-3′, in which the RARE sequence is underlined.

Some of the reporter constructs containing regions of the *PHOX2A* promoter have been previously described by Flora et al. [5]. However, as these regions were cloned into the pGL3basic vector backbone and this was unspecifically transactivated by ATRA treatment, we re-cloned fragments of the human *PHOX2A* 5′-flanking region into the ATRA-unresponsive pGL4basic plasmid (Promega) upstream of the *Firefly* luciferase gene. Details concerning the plasmid construction are available in the Supplementary Material. The *PHOX2A* expression vector was obtained by cloning human *PHOX2A* cDNA [7] into the EcoRI site of pCMV-myc (Clontech Laboratories Inc., Mountain View, CA, USA). All of the constructs were checked by means of restriction analysis and partial sequencing.

### 2.8 Transfections and luciferase assays

The transfection experiments were performed by means of lipofection as described by Flora et al. [37] using 1.6×10^5^ SK-N-BE(2)C cells. Luciferase was assayed using the Dual Luciferase Reporter Assay System as previously described [5], [38].

### 2.9 Protein preparation, immunoprecipitation and western blot analysis

The total protein extracts were obtained from sub-confluent cells using the freeze and thaw method as described by Benfante et al. [33] and the nuclear extracts were prepared as previously described [35].

For immunoprecipitation, SK-N-BE(2)C cells were harvested after being treated with ATRA and MG-132 as described in the by means of centrifugation at 7000 rpm for 5 min. The pellet was resuspended in 150 µl lysis buffer containing non-ionic detergent (1% Triton X-100, 50 mM Tris–HCl, pH 7.5, 150 mM NaCl, 2 mM EDTA, 0.5 mM DTT, 0.2 mM PMSF, and the Sigma-Aldrich protease inhibitors cocktail), and incubated for one hour at 4 °C on a rotating wheel. The extracts were then clarified by means of 30 min centrifugation at 13,200 rpm/4 °C, and pre-cleared using protein G/agarose bead slurry (InVitrogen) and chicken pre-immune IgY (Santa Cruz Biotechnology, Inc., Dallas, Texas, USA). Three milligrams of the pre-cleared extracts were incubated overnight at 4 °C with 5 μg polyclonal chicken anti-PHOX2A antibody (Davids Biotechnologie), or pre-immune chicken IgY (Santa Cruz Biotechnology) and the immunocomplexes were captured by protein G/agarose bead slurry (InVitrogen). Because of the poor binding of chicken antibodies to protein G, a bridging antibody (rabbit anti-chicken IgG, Upstate, Lake Placid, NY, USA) was added to enhance the capture of the immunocomplexes. The beads were collected by means of centrifugation, gently washed, and resuspended in sample loading buffer, and the immunocomplexes were dissociated from the beads by boiling the samples.

The proteins were separated by means of 10% SDS-PAGE and transferred to a nitrocellulose membrane (Schleicher & Schuell BioScience GmbH, Dassel, Germany), and Western blotting was carried out as previously described [7] using the chicken anti-PHOX2A [7], mouse monoclonal anti-PHOX2B (B-11), rabbit anti-Sp1 (Santa Cruz Biotechnology), mouse anti-β-tubulin (Sigma-Aldrich) and mouse anti-Ubiquitin (BIOMOL GmbH, Hamburg, Germany) as primary antibodies; the secondary antibodies (rabbit anti-chicken, Davids Biotechnologie; goat anti-rabbit and anti-mouse, Pierce Biotechnology Inc., Rockford, ME, USA) were conjugated with horseradish peroxidase. The bands were revealed using Super Signal West Dura (Pierce Biotechnology Inc.), with standard molecular weights (New England Biolabs Inc., Beverly, MA USA) being loaded in parallel.

### 2.10 Data analysis

NIH Image 1.61/fat software was used for the densitometric analysis of the signals obtained from the Western blots. The results are given as the mean values of at least three independent experiments, and standard deviation (SD) or standard error (SEM) as indicated in the The data were analysed by means of a paired two-tailed Student's *t* test or one-way ANOVA using GraphPad Prism 5 Software (GraphPad Software, Inc.) as described in the; *p* values of <0.05 were considered significant.

## 3 Results

### 3.1 Effects of ATRA on PHOX2A and PHOX2B expression

The effect of ATRA on the expression of *PHOX2A* and *PHOX2B* was studied using the SK-N-BE(2)C NB cell line, a very well-characterised model of RA-induced neuronal differentiation [39]. Its differentiation potential was tested by treating the cells with 10 μM ATRA for different periods of time, evaluating their morphology and the differential expression of some target genes, and measuring the responsiveness of a reporter construct in which transcription was driven by the RARE. Phase-contrast microscopy showed that the morphology of the SK-N-BE(2)C cells started changing after 24 h of ATRA exposure (Fig. S1A, panel b), and neurite outgrowth was clearly evident after 72 h (Fig. S1A, panel c); exposure to the vehicle alone did not affect their morphology at any time (Fig. S1A, panels d and e), leaving them indistinguishable from the control (Fig. S1A, panel a).

At molecular level, ATRA-induced SK-N-BE(2)C cells differentiation is characterised by changes in the expression of some target genes, including *HASH1* [40], [41], and Northern blot analysis of RNA from the cells treated with ATRA for different times revealed a unique transcript of approximately 3 Kb encoding HASH1 (Fig. S1B, lanes 1, 2 and 4), which was observed in all of the tested NB cells, whereas no signal was observed in the HeLa cells (Fig. S1B, lane 3). After 24 h exposure to ATRA, the expression of the transcript became barely detectable (Fig. S1B, lane 5) and remained very low for up to 96 h (Fig. S1B, lanes 6–8). This is in line with the findings of Söderholm et al. [41] and, together with the morphological data, indicates that the most relevant biological responses of neuroblastoma cells to ATRA exposure were completely reproducible under our experimental conditions.

The responsiveness of SK-N-BE(2)C cells to ATRA was also tested by means of the transient transfection of a reporter construct in which the transcription was driven by the RARE identified in the promoter of the *β2* subunit of retinoic acid receptor (β-RARE luc), cloned upstream of SV40 promoter. Fig. S1C shows that the SK-N-BE(2)C cells were fully responsive to ATRA treatment as the expression of the luciferase reporter gene increased 40-fold over that of untreated cells (compare lanes 1 and 2). Treatment with vehicle alone had no effect on the luciferase (Fig. S1C, lane 3). All of these data further confirm that the most relevant biological responses of SK-N-BE(2)C cells to ATRA exposure were completely reproducible under our experimental conditions.

**Fig. 1:**
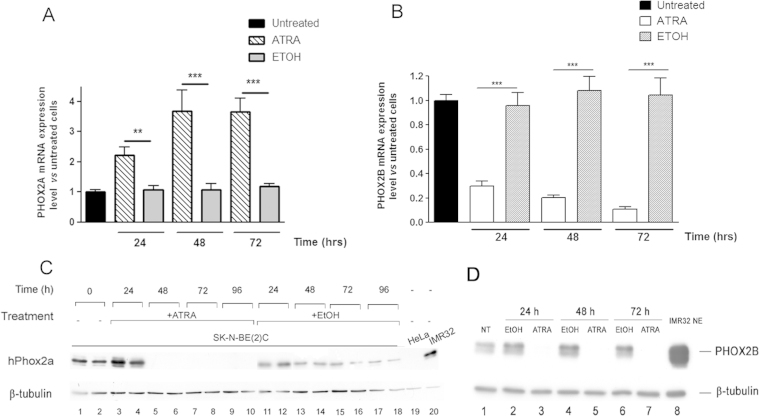
Effects of ATRA on PHOX2A and PHOX2B expression in SK-N-BE(2)C cells. (A and B) qPCR analyses of PHOX2A (panel A) and *PHOX2B* expression (panel B). SK-N-BE(2)C cells were treated with ATRA for the indicated times, and gene expression was determined by means of real-time PCR using the *GAPDH* gene as an internal control. The bars represent the mean values±SD of three independent experiments expressed as fold-induction in comparison with the untreated sample (black bars). Hatched (panel A) and empty (panel B) bars=treated cells; grey bars=vehicle-treated samples. **p<0.01; and ***p<0.001 indicate statistically significant differences in *PHOX2A* and PHOX2B mRNA expression between the vehicle treated (grey bars) and the cells treated with ATRA for different periods of time (one-way ANOVA, post-Tukey's test). (C and D) Western blots of total protein extracts obtained from SK-N-BE(2)C cells treated with ATRA 10 μM (lanes 3–10 in panel C, and lanes 3, 5 and 7 in panel D) or ethanol (lanes 11–18 in panel C and 2, 4 and 6 in panel D) for different periods of time. The filter was probed with the anti-PHOX2A (panel C) or anti-PHOX2B antibody (panel D). The membranes were probed with an antibody against β-tubulin for normalisation purposes. The negative and positive controls were protein extracts of HeLa (panel C, lane 19) and IMR32 cells (panel C, lane 20, and panel D, lane 8). Lanes 1 and 2 (panel C), and lane 1 (panel D): untreated cells.

Fig. 1A shows that the *PHOX2A* transcript [5] was considerably induced after 24 h exposure to ATRA, and this level of expression increased for up to 72 h (Fig. 1A, hatched *vs* grey bars), whereas exposure to the vehicle alone did not affect *PHOX2A* expression at any time (Fig. 1A, grey bars *vs* black bar). The induction of *PHOX2A* mRNA by RA was reproducible and statistically significant. On the contrary, *PHOX2B* transcript expression decreased by 70% after 24 h exposure, and by up to 90% after 72 h (Fig. 1B, white *vs* grey bars).

Western blot analysis was used to evaluate whether the induction of *PHOX2A* mRNA and reduction in *PHOX2B* mRNA expression was paralleled by an adequate increase/decrease in the corresponding protein. Fig. 1C shows that, after 24 h treatment with ATRA, there was a substantial and statistically significant increase in PHOX2A protein levels (Fig. 1C, lanes 3 and 4 *vs* control lanes 1 and 2); however, surprisingly, no trace of the protein was found after 48 h and for up to 96 h (Fig. 1C, lanes 5–10). On the contrary, PHOX2B protein was not detectable after no more than 24 h ATRA treatment (Fig. 1D, lanes 3, 5 and 7 *vs* lanes 2, 4 and 6).

### 3.2 ATRA acts at transcriptional level

**Fig. 2:**
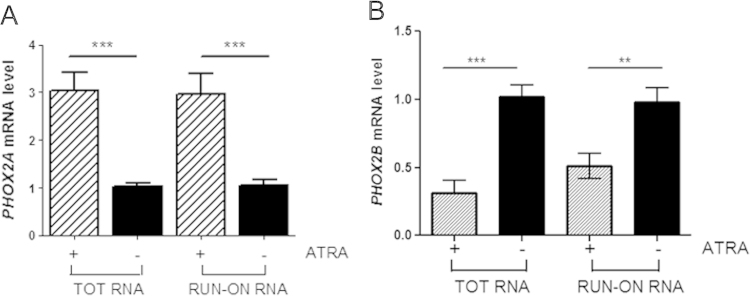
Effects of ATRA on the transcription of *PHOX2A* and *PHOX2B* mRNA. (A and B) Run-on analysis. The SK-N-BE(2)C cells were treated with ATRA for 24 h, and *PHOX2A* (panel A) and *PHOX2B* gene expression (panel B) was determined by means of real-time PCR using the *GAPDH* gene as an internal control. The bars represent the mean values±SD of three independent experiments using total and nuclear run-on RNA in the treated cells (striped bars), expressed as fold-induction over the vehicle-treated samples (black bars). **p<0.01 and ***p<0.001 (one-way ANOVA, post-Tukey's test).

We used nuclear run-on experiments to investigate whether ATRA acts directly at transcriptional level. Fig. 2A and B (TOT RNA) confirmed that 24 h ATRA exposure induced a three-fold increase in *PHOX2A* and three-fold decrease in *PHOX2B* mRNA expression, and the quantitative run-on experiments showed that this changes were due to the new induction or repression of transcription caused by the drug (Fig. 2A and B, RUN-ON RNA). In brief, ATRA increased *PHOX2A* and decreased *PHOX2B* mRNA levels by acting on their transcription.

### 3.3 Mapping the retinoic acid responsive elements (RAREs)

The nuclear run-on analyses (Fig. 2) showed that the changes in *PHOX2A* and *PHOX2B* relative expression was mainly due to a transcriptional mechanism. A computer-assisted analysis of 10 Kb of the *PHOX2A* promoter sequence using MatInspector software (www.genomatix.de) revealed the presence of putative RAREs, which may explain the increased *PHOX2A* expression induced in the SK-N-BE(2)C cell line by ATRA treatment.

**Fig. 3:**
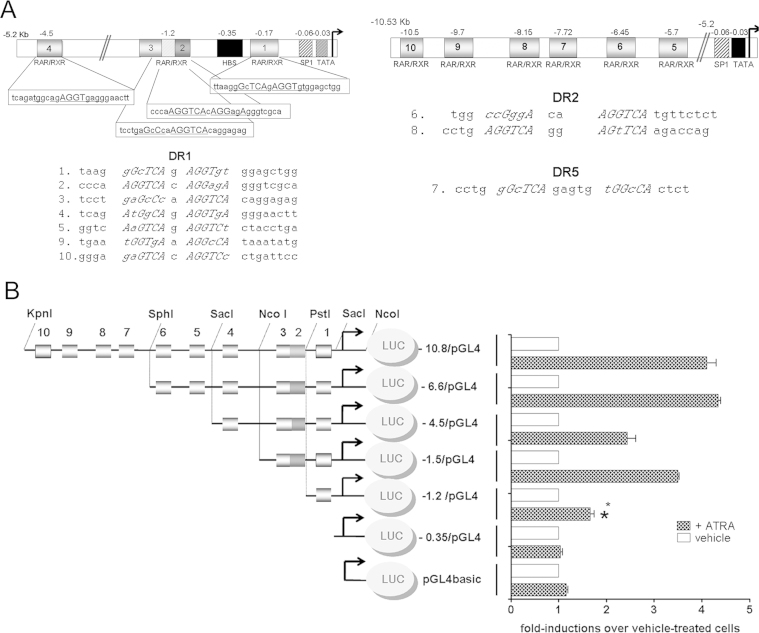
Functional mapping of *PHOX2A* retinoic acid responsive elements. (A) Schematic illustration of the putative RAREs identified in the *PHOX2A* promoter region. *Left:* The region spanning 5 Kb upstream of the *PHOX2A* transcriptional start site. *Right:* The region spanning nucleotides −10,530 to −5200 of the *PHOX2A* 5′-flanking region. The consensus RARE is also indicated. (B) Left: Schematic illustration of the constructs. The boxes represent the putative RARE sequences #1-#10, as assessed by means of computer-assisted analysis (Genomatix, MatInspector), and the arrows the transcription start site. The figure shows the restriction sites used to clone the different parts of the *PHOX2A* 5′-flanking region; the grey oval represent the *Firefly* luciferase reporter gene (luc). Right: Luciferase assays. SK-N-BE(2)C cells were transiently transfected with the constructs shown on the left, and treated with ATRA for 24 h before luciferase assay. The bars show the transcriptional activity of the constructs expressed as fold-inductions over vehicle-treated cells (mean values±SD of at least three independent experiments performed in triplicate). The asterisk indicates a statistically significant difference between the cells treated with ATRA or vehicle alone for the same period of time (Student's *t* test. *p<0.05).

Ten putative RAREs were identified, two of which (#2 and #3) partially overlapped. Most of the RAREs were directed repeats separated by one nucleotide (DR1), two were DR2 (#6 and #8) and only one was a DR5 (#7), although none of them perfectly matched the consensus (Fig. 3A). In order to test whether these sites were functionally responsive to RA, we generated a series of constructs spanning the *PHOX2A* promoter from position −10.53 Kb to position −0.35 Kb, and performed transient transfections. The KpnI-NcoI fragment (Fig. 3B, −10.8/pGL4), which contains all ten RAREs, and the SphI-NcoI fragment (Fig. 3B, −6.6/pGL4), which contains RAREs 1–6, responded to ATRA treatment with an almost five-fold induction of construct activity in comparison with the vehicle-treated cells. The SacI-NcoI fragment (Fig. 3B, −4.5/pGL4), which spans 5.2 Kb of the promoter upstream of the transcriptional start site and contains RAREs 1–4, was also responsive, although to a lesser extent (Fig. 3B). However, further deletion of RARE 4 (construct −1.5/pGL4) did not affect the activity of the *PHOX2A* promoter, which remained fully responsive to ATRA. The −1.2/pGL4 construct containing only the RARE 1 sequence also remained responsive, and showed a statistically significant two-fold increase in promoter activity in comparison with the vehicle-treated cells, whereas the deletion of RARE 1 (Fig. 3B, −0.35/pGL4) completely abolished the ability of ATRA to induce *PHOX2A* promoter activity.

These data indicate that the responsiveness to ATRA is a result of the contribution of the three RARE sequences located in the first 1.5 Kb of the *PHOX2A* promoter.

On the other hand, *in silico* analysis of the *PHOX2B* promoter did not reveal the presence of canonical RARE elements, thus suggesting that the down-regulation of *PHOX2B* expression is not mediated by the direct binding of RA receptors to the promoter, and that other transcription factors mediate the effect of RA on *PHOX2B* gene expression.

### 3.4 Down-regulation of PHOX2B expression is not due to a direct effect of PHOX2A

Control of the temporal and spatial expression of *PHOX2A* and *PHOX2B* is fundamental during the specification of neuronal identity, and many studies have tried to elucidate the exact molecular mechanisms involved in regulating their expression over the last few years. We have previously demonstrated that PHOX2B regulates the transcription of the *PHOX2A* gene by directly binding and transactivating its promoter [5]. We have also characterised the *PHOX2B* promoter, and demonstrated by means of biochemical and functional assays that most of its transcriptional activity is sustained by auto-regulatory mechanisms involving PHOX2B binding and transactivation [4].

Chromatin immunoprecipitation (ChIP) assays show that PHOX2A also participates in the transcriptional complex assembled on the *PHOX2B* promoter [4] (Fig. 2S, panels A and B). The *PHOX2B* promoter has five binding sites for homeodomain proteins (Fig. S2D), and EMSA analysis (Fig. 2S, panel C) showed that PHOX2A is also capable of binding the ATTA2 (Fig. 2S panel C, lanes 7–10) and ATTA3 (Fig. 2S panel C, lanes 12–15) sites in the *PHOX2B* promoter; moreover, unlike PHOX2B, PHOX2A, binds also the ATTA1 and ATTA5 sites (Fig. 2S, panel C, lanes 2–5 and 22–25), although with lower affinity.

**Fig. 4:**
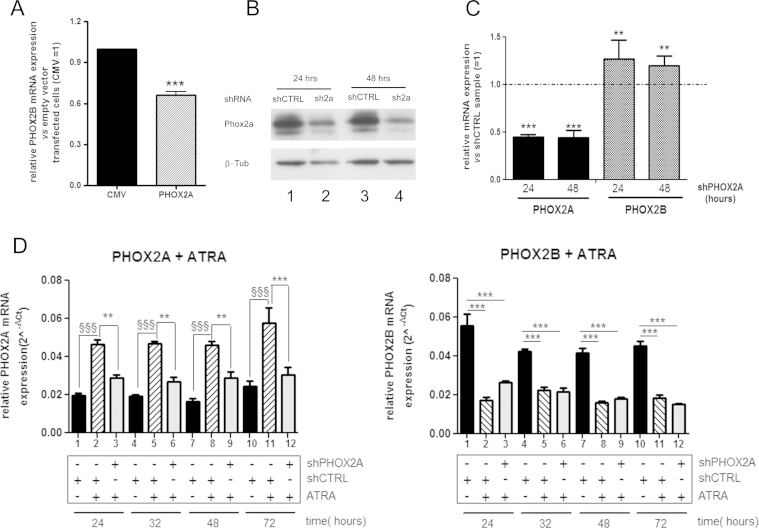
PHOX2A induction does not mediate the ATRA-induced down-regulation of *PHOX2B* expression. (A) qPCR analysis of *PHOX2B* expression following PHOX2A over-expression. SK-N-BE(2)C cells were transfected with PHOX2A cDNA expression vector (striped bar), and *PHOX2B* gene expression was determined by means of real-time PCR using the *GAPDH* gene as an internal control. The bars represent the mean values±SD of three independent experiments expressed as fold-inductions in comparison with the cells transfected with empty vector (black bar). ***p<0.001 indicates statistically significant differences in *PHOX2B* mRNA expression (Student's *t* test). (B) Western blots of PHOX2A silencing. SK-N-BE(2)C cells were transfected with the shRNA construct targeting the 3′-UTR of *PHOX2A* (sh2a, lanes 2 and 4) or scrambled shRNA (shCTRL, lanes 1 and 3). PHOX2A protein levels were determined 24 (lanes 1 and 2) and 48 h after transfection (lanes 3 and 4), using an anti-PHOX2A antibody. The membrane was probed with an antibody against β-tubulin for normalisation purposes. (C) qPCR analysis of *PHOX2A* (black bars) and *PHOX2B* expression (grey bars) upon PHOX2A silencing. SK-N-BE(2)C cells were transfected with the shRNA construct targeting the 3′-UTR of *PHOX2A* (shPHOX2A) and gene expression was determined 24 and 48 h after transfection by means of real-time PCR using the *GAPDH* gene as an internal control. The bars represent the mean values±SD of at least three independent experiments, expressed as fold differences in comparison with the cells transfected with scrambled shRNA (shCTRL=1). **p<0.01 and ***p<0.001 indicate statistically significant differences in gene expression relative to scramble transfected cells. (D) *PHOX2A* and *PHOX2B* mRNA expression upon ATRA treatment after PHOX2A silencing. SK-N-BE(2)C cells were transfected with *PHOX2A* shRNA (shPHOX2A, grey bars) or scrambled shRNA constructs (shCTRL, striped and black bars), and treated with 10 µM ATRA (grey and striped bars) for the indicated times. *PHOX2A* (left panel) and *PHOX2B* (right panel) gene expression was determined by means of real-time PCR using the *GAPDH* gene as an internal control. The bars represent the mean values±SD of three independent experiments, expressed as relative mRNA levels calculated using the 2^−ΔCt^ method. Left panel: **p<0.01 and ^§§§^*p*<0.001 (one-way ANOVA, post-Tukey's test) indicate statistically significant differences in *PHOX2A* mRNA expression between the cells transfected with scrambled shRNA (striped bars) and the cells transfected with shPHOX2A (grey bars) treated with ATRA or between the cells transfected with scrambled shRNA treated with ATRA (striped bars) or vehicle (black bars). There was no statistically significant difference between the vehicle- and ATRA-treated cells respectively transfected with shCTRL (black bars) and with shPHOX2A (grey bars). Right panel: ***p<0.001 indicate statistically significant differences in *PHOX2B* mRNA expression between the ATRA- and vehicle-treated cells transfected with shCTRL (bars 2, 5, 8 and 11 *vs* 1, 4, 7 and 10) or shPHOX2A (bars 3, 6, 9 and 12 *vs* 1, 4, 7 and 10). There was no statistically significant difference between the ATRA-treated cells transfected with shPHOX2A (grey bars) and those transfected with shCTRL (striped bars).

We then asked whether the down-regulated expression of *PHOX2B* was related to, and mediated by the increased expression of PHOX2A induced by ATRA treatment. The over-expression of PHOX2A in the SK(2)C NB cell lines showed a statistically significant 40% reduction in the expression of endogenous *PHOX2B* (Fig. 4A, hatched *vs* black bar), thus suggesting that PHOX2A negatively modulates *PHOX2B* expression, although the reduction was not as great as that observed after an ATRA challenge. These data were confirmed by silencing PHOX2A expression for 24 and 48 h (Fig. 4B, lanes 2 and 4 *vs* 1 and 3), and an approximately 50% reduction in PHOX2A expression (Fig. 4C, black bars) corresponded to a slight, but statistically significant increase in *PHOX2B* expression (Fig. 4C, grey bars). In order to investigate whether the down-regulation of *PHOX2B* induced by ATRA treatment was at least partially due to an increase in PHOX2A expression, we counteracted the ATRA-induced increase in *PHOX2A* expression in SK(2)C cells transfected with *PHOX2A* shRNA, and measured the corresponding level of *PHOX2B* mRNA. Fig. 4D (left panel) shows that the presence of *PHOX2A* shRNA blocked the ATRA-induced *PHOX2A* expression (grey *vs* hatched bars) for up to 72 h to an extent that was not statistically different from that observed in the samples transfected with a scrambled shRNA (shCTRL) and not challenged to ATRA treatment (grey *vs* black bars). However, the reduction in *PHOX2B* expression was not affected by the presence of the *PHOX2A* shRNA (Fig. 4D, right panel; grey *vs* hatched bars) at any time. These data suggest that PHOX2A and PHOX2B cross-regulate their own expression, but this mechanism is not involved in regulating ATRA-induced PHOX2B down-regulation.

### 3.5 ATRA reduces PHOX2A protein half-life by means of proteasomal degradation

**Fig. 5:**
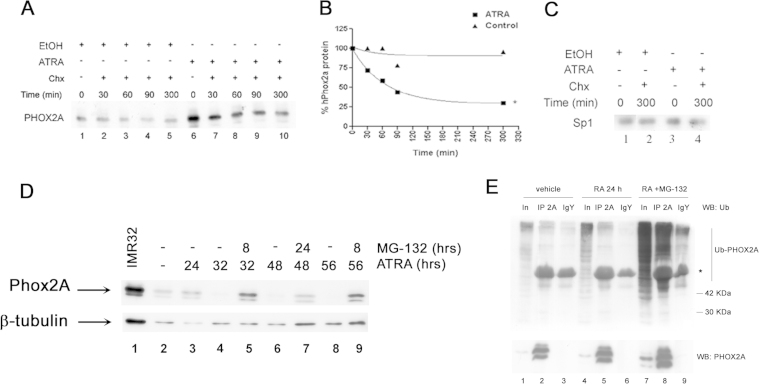
ATRA induces the proteasome-mediated degradation of PHOX2A protein. (A) SK-N-BE(2)C cells exposed to ethanol (lanes 1–5) or 10 μM ATRA (lanes 6–10) for 24 h were treated with cycloheximide at a final concentration of 10 μg/ml. The total protein extracts were obtained after treatment with cycloheximide for the indicated periods of time, and analysed by means of Western blotting. (B) Densitometric signal quantification expressed as the percentage of PHOX2A expression after 24 h exposure to ethanol (triangles) or ATRA (squares). The asterisk indicates a statistically significant difference between the control and ATRA-treated cells after 300 min (Student's *t* test *p*<0.05). *C*) The same protein extracts as those used in the experiment shown in panel A (lanes 1, 5, 6 and 10) were analysed for the expression of the Sp1 transcription factor by means of Western blotting. (D) Western blots of extracts from SK-N-BE(2)C cells treated with ATRA for 24 h (lane 3), 32 h (lane 4), 48 h (lanes 6), and 56 h (lane 8), alone or in combination with the simultaneous treatment with the proteasome inhibitors MG-132 for eight (lanes 5 and 9) and 24 h (lane 7). The positive controls were untreated SK-N-BE(2)C cells at time 0 (lane 2), and 10 μg of nuclear extract from IMR32 cells (lane 1) *E*) Total extracts from SK-N-BE(2)C cells treated with EtOH (vehicle, lanes 1–3) or 10^−5^ ATRA for 24 h (lanes 4–6), followed by simultaneous treatment with MG-132 for eight hours (lanes 7–9), were immunoprecipitated with anti-PHOX2A antibodies (IP2A, lanes 2, 5, 8), and the level of ubiquitination was evaluated by means of Western blotting using an anti-ubiquitin (Ub) antibody (upper panel). Immunoprecipitation with chicken pre-immune IgY was used as a control, (IgY, lanes 3, 6 and 9). Lanes 1, 4 and 7 show pre-immunoprecipitation total extract [10% input (In)]. The same membranes were stripped and re-probed with anti-PHOX2A antibody (lower panel). The asterisk indicates an specific band due to IgY heavy chain. Ub-PHOX2A: Ubiquitin-PHOX2A conjugates.

As shown in Fig. 1A, 24 h ATRA treatment led to a substantial and statistically significant increase in PHOX2A expression; however, surprisingly, no trace of the protein was found after 48 h exposure and for up to 96 h (Fig. 1A). In order to investigate whether the delayed effects of ATRA on PHOX2A protein expression correlated with decreased protein stability, we evaluated PHOX2A protein half-life by treating SK-N-BE(2)C cells with ATRA or ethanol for 24 h, followed by 10 μg/ml cycloheximide for different time periods (Fig. 5A). The half-life of the protein was significantly reduced (50%) in comparison with the levels observed in the cells exposed to the vehicle alone (Fig. 5A, lanes 2–5 *vs* lanes 7–10) after no more than 90 min (Fig. 5B).

In order to confirm that the effects of ATRA on protein stability were PHOX2A specific, we studied the expression of the transcription factor Sp1 after 300 min exposure to RA in the same samples as those used in Fig. 5A: Fig. 5C shows that the presence of RA had no effect on Sp1 expression.

In order to investigate whether the reduced stability of PHOX2A protein was due to increased proteasome-mediated degradation, SK-N-BE(2)C cells were treated with MG-132, an inhibitor of proteasomal activity. The cells were exposed to MG-132 for the last eight hours of a 32-h exposure period, and this increased the amount of PHOX2A protein in comparison with the amount induced by ATRA alone (Fig. 5D, lane 5 *vs* lane 4). The simultaneous presence of the inhibitor for up to 24 h (a total of 48 h of ATRA treatment; Fig. 5D, lane 7), or for the last eight hours of a 56 h exposure to ATRA (Fig. 5D, lane 9) rescued PHOX2A protein expression from degradation (compare lanes 7 with 6 and lanes 9 with 8). Furthermore, PHOX2A immunoprecipitation and Ubiquitin Western blotting revealed that PHOX2A-Ubiquitin conjugates are increased by treatment with ATRA combined with MG-132 than treatment by ATRA alone (Fig. 5E, lane 8 *vs* lane 5), thus confirming that the disappearance of PHOX2A protein is due to selective proteasomal degradation, whereas the mRNA remains stably expressed.

## 4 Discussion

We investigated the effects of ATRA on the expression of *PHOX2A*, a candidate tumour suppressor gene [14], using the undifferentiated SK-N-BE(2)C human NB cell line as a model, and found that they were apparently opposite: it initially acted as a positive regulator of gene expression, but later triggered a process that culminated in the complete disappearance of the transcription factor. The positive effects of ATRA were mainly due to direct stimulation of gene transcription by means of the contribution of three RARE sequences located in the first 1.5 Kb of the *PHOX2A* promoter, but the identity of the RA nuclear receptor isoforms involved in this regulation is still unknown.

The increase in PHOX2A protein product during the first 24 h of treatment, followed by its down-regulation after 48 h even though the expression of *PHOX2A* mRNA remained constantly up-regulated, led us to hypothesise that ATRA might promote the disappearance of PHOX2A protein by means of two different molecular mechanisms: translation inhibition and/or increased protein degradation. Our data strongly suggest that the proteasome plays a pivotal role, and raise the question as to which pathways link ATRA exposure to PHOX2A degradation.

RA down-regulates cell proliferation and promotes neurogenesis by arresting cells in the G1 phase of the cell cycle [31]. Although the underlying mechanism is still unclear, it is known that RARβ is a necessary component of the inhibitory effects of ATRA on the growth of NB cells [42]; furthermore, RA induces cell-cycle arrest in G1 by decreasing the cyclin-dependent kinase (Cdk) activity required for the G1/S transition and the accumulation of the p27^Kip1^Cdk inhibitor (CKI) [43], [44] that impair growth and activate the differentiation programme. The stability of p27^Kip1^ is regulated by the E3 ubiquitin ligase SKP2, and RA stabilises p27^Kip1^ by enhancing the proteasome-mediated degradation of SKP2 in a number of cancer cell lines. Like PHOX2A, SKP2 protein disappears, but its mRNA persists during the RA treatment of SH-SY5Y cells, thus suggesting that RA post-transcriptionally regulates SKP2 [45]. Ballas et al. and Singh et al. [46], [47] have shown that REST, a key regulator of neuronal genes during neuronal differentiation, is regulated by ATRA in a manner that is similar to the regulation of PHOX2A, and suggested that the degradation of REST may facilitate a rapid transition to terminal differentiation; given that REST is mainly a negative regulator, this permits the differential expression of a subset of genes with lower-affinity REST binding sites. These findings suggest that regulation of proteasome-mediated degradation of the proteins involved in different aspects of cell metabolism may be a common mechanism by means of which RA controls the order of the signalling events necessary for a cell's response to retinoid-induced differentiation.

Like *PHOX2A, PHOX2B*, is overexpressed in a number of tumours and NB cell lines. However, there are conflicting hypotheses concerning the significance of this in the pathogenesis of NBs. Raabe et al. [16] suggest that *PHOX2B* up-regulation is simply a marker of tumour lineage and not a contributor to malignant phenotype, given that neuroblastoma arise at a time when *PHOX2B* is normally expressed during neurodevelopment, and its forced overexpression decreases proliferation. Furthermore, they rule out PHOX2A involvement in the pathogenesis of NBs because no mutations have been found in the PHOX2A coding region [14], [16]. As the expression of PHOX2B precedes that of PHOX2A during development, and *in vitro* experiments have shown that the forced over-expression of PHOX2B regulates *PHOX2A* [5], [6], it is possible to speculate that *PHOX2A* up-regulation may be due to a high level of PHOX2B expression, because silencing *PHOX2B* in NB cell lines leads to the down-regulation of *PHOX2A* [22].

However, in contrast to data described in Bachetti et al. [22], our data suggest a new mechanism cross-regulating PHOX2A and PHOX2B as they show that PHOX2A negatively modulates *PHOX2B* expression. This discrepancy may be due to differences of cell system (SK-N-BE(2)C cells as opposed to SH-SY5Y and IMR32 cell lines), and so the up-regulated expression of *PHOX2A* and *PHOX2B* in NBs might be the result of cross-regulation.

Conversely, the observation that the proliferation of undifferentiated PHOX2B^+^ neuronal progenitors promotes NB cell proliferation and stemness indicates that PHOX2B is a critical regulator in the pathogenesis of NBs [23]. It is not known what decides whether PHOX2B is pro- or anti-proliferative, but it can be hypothesised that the effects of *PHOX2B* over-expression depend on cell context.

As we did not map any RARE in the *PHOX2B* promoter, we wondered whether the *PHOX2B* down-regulation we observed during RA-driven differentiation was due to the up-regulation of PHOX2A, which down-regulates the expression of *PHOX2B*. However, by silencing endogenous *PHOX2A* expression, we found that *PHOX2B* regulation by PHOX2A is not the mechanism underlying the effect of RA. The association between MYCN amplification (an important prognostic factor in NB) and *PHOX2B* expression in human NB cell lines [23], the down-regulation of MYCN expression after an RA challenge [48], [49], the up-regulation of PHOX2B associated with MYCN over-expression, and the down-regulation of PHOX2B and Mash1 when siRNA is used to inhibit MYCN [23], all support the hypothesis that a high *PHOX2B* expression level is due to the direct regulation of *PHOX2B* by MYCN, and we are currently investigating whether the down-regulation of *PHOX2B* is related to a reduction in MYCN expression.

In any case, our findings highlight the importance of the regulation of gene dosage in the processes driving final cells differentiation. The action of ATRA on PHOX2A and PHOX2B expression may not only determine a cell's fate, but also stabilise it by maintaining lineage-specific expression patterns, as confirmed by the observation that PHOX2B is down-regulated during final neuronal differentiation [13]. The dysregulation of one of these pathways (perhaps leading to the accumulation of PHOX2B^+^ progenitor cells) may be one of the major mechanisms involved in the pathogenesis of NBs.

The down-regulation of *PHOX2B* expression induced by ATRA supports the idea that *PHOX2B* up-regulation is due to a block in the differentiation process, and so its down-regulation may force the differentiation of NB cells. Experiments aimed at identifying drugs capable of reducing *PHOX2B* expression in the IMR32 cell line [50] have not found that ATRA has any effect, and this is in line with our unpublished findings showing that *PHOX2A* and *PHOX2B* are not regulated by ATRA in IMR32 cells, although the cells are ATRA responsive as measured on the basis of RARE reporter vector activation (data not shown). However, the SH-SY5Y NB cell line responds to ATRA in a similar manner ([33] and data not shown), albeit with different kinetics, thus confirming that the effects of ATRA on *PHOX2A* and *PHOX2B* expression are not peculiar to the SK-N-BE(2)C cell line but may reflect a general mechanism that could be important in retinoid-induced neuronal differentiation.

These observations reinforce the idea that PHOX2A and PHOX2B may be useful biomarkers for NB staging, prognosis and treatment decision making [13].

These findings and other published data [51], [52] shed new light on the pathways driving undifferentiated and proliferating cells to activate a differentiation programme by regulating the activation/deactivation of transcription factors such as PHOX2A and PHOX2B, which control cell-specific genes such as the *DβH* and cell cycle regulators such as *p27*^*kip1*^. In particular, the work of Andrisani's lab. [52] has shown that PHOX2A is intrinsically programmed to be active for a defined period of time, although in our case it is not the activity but the presence of PHOX2A that is temporally regulated by ATRA. A better understanding of the molecular mechanisms underlying this control may provide new opportunities for the development of new drugs that can be used in cancer therapy.

## 5 Conclusions

In conclusions**,** our findings demonstrate that PHOX2A expression is finely controlled during retinoic acid differentiation and suggest that, together with PHOX2B down-regulation, they may be useful biomarkers for NB staging, prognosis and treatment decision making.

## Footnotes

Appendix A Supplementary data associated with this article can be found in the online version at doi:10.1016/j.yexcr.2016.02.014.
