# Supplementary material for: PHOX2A and PHOX2B are differentially regulated during retinoic acid-driven differentiation of SK-N-BE(2)C neuroblastoma cell line
Source: Exp Cell Res. 2016 Mar 1;342(1):62–71. doi: 10.1016/j.yexcr.2016.02.014 (PMC4819706; doi:10.1016/j.yexcr.2016.02.014)
Supplement: Supplementary file 1 — Supplementary material [file mmc1.docx]

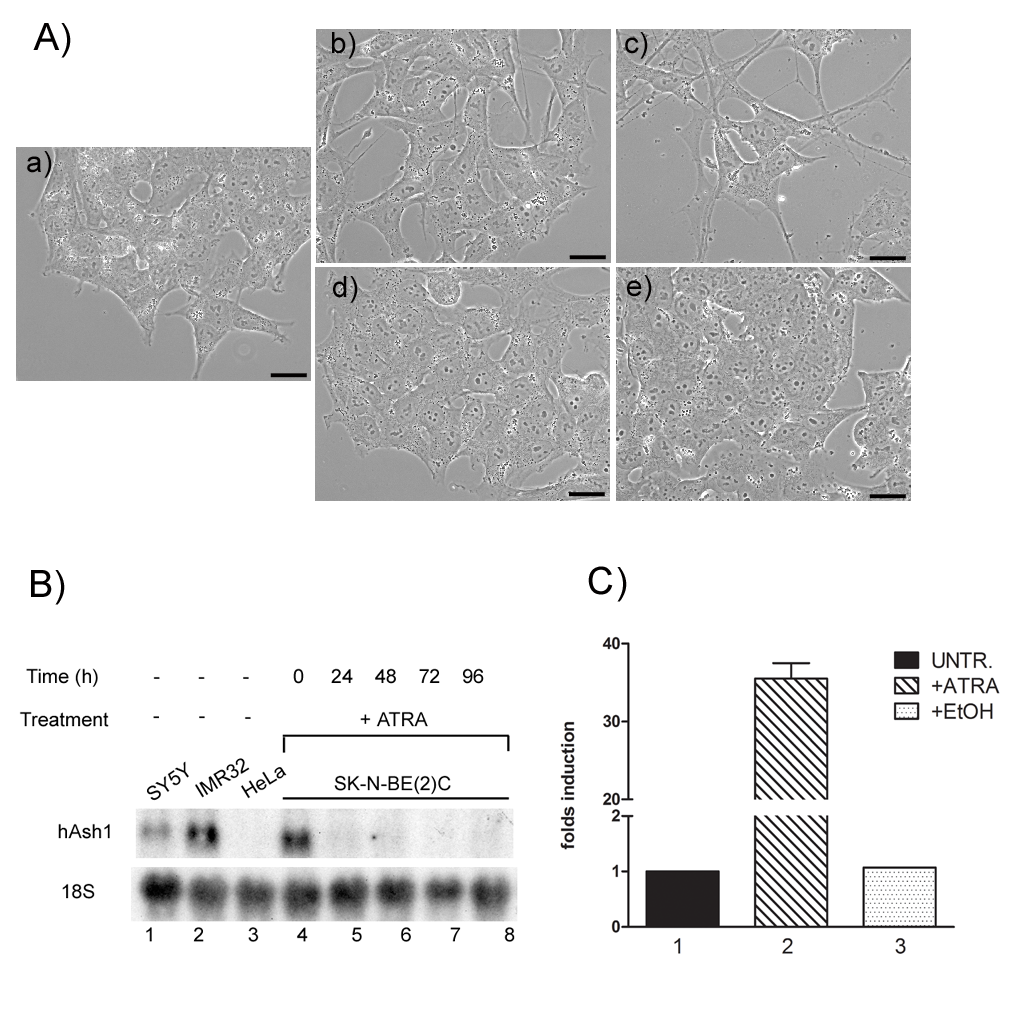


**Fig. S1.** Differentiation of ATRA-treated SK-N-BE(2)C cells. A) Phase contrast microscopy images of SK-N-BE(2)C cells. *Panel a*: untreated cells; *panels b and c*: cells exposed to 10 μM ATRA for respectively 24 and 72 hours; *panels d and e*: cells exposed to the vehicle alone (100% ethanol) for respectively 24 and 72 hours. The bars correspond to 20 μm. B) Northern blot analysis. Ten micrograms of total RNA purified from the indicated cell lines (lanes 1-3), or SK-N-BE(2)C cells treated with 10µM ATRA for different periods of time (lanes 4-8), were hybridised with the *HASH1* cDNA probe. C) SK-N-BE(2)C cells were transiently transfected with the β-RARE-luc reporter construct, and then treated with ATRA (striped column) or vehicle (grey column) for 24 hours. The columns show the transcriptional activity of the construct as fold increases over the activity of the untreated cells (black column, set as 1). The data represent the mean values ± SD (error bars) of at least three independent experiments carried out in triplicate.
