# Supplementary material for: PHOX2A and PHOX2B are differentially regulated during retinoic acid-driven differentiation of SK-N-BE(2)C neuroblastoma cell line
Source: Exp Cell Res. 2016 Mar 1;342(1):62–71. doi: 10.1016/j.yexcr.2016.02.014 (PMC4819706; doi:10.1016/j.yexcr.2016.02.014)
Supplement: Supplementary file 2 — Supplementary material [file mmc2.docx]

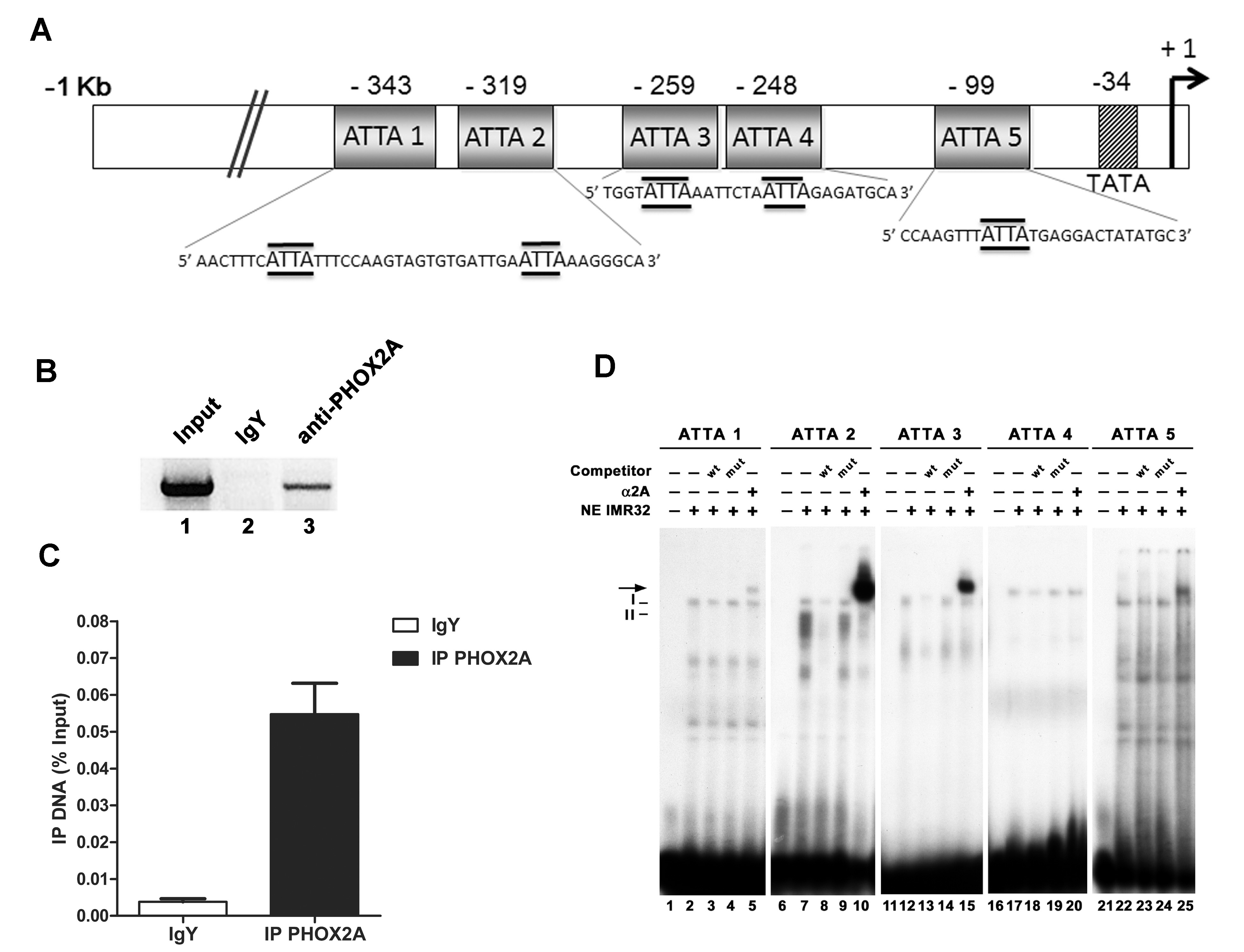


**Fig. S2: PHOX2A binds the PHOX2B promoter.** A) Schematic representation of the 1 Kb PHOX2B promoter region containing the five “ATTA” sites [4]. B and C) Chromatin immunoprecipitation. Chromatin was immunoprecipitated from IMR32 cells using anti-PHOX2A antibodies (panel B, *lane 3*); pre-immune IgY was used as the negative control (panel B, *lane 2*). The input (panel B, *lane 1*) represents 1% of total chromatin extract. The precipitated DNA fragments were amplified by means of a set of primers bordering the five “ATTA” sites in the *PHOX2B* promoter, which led to the generation of PCR products of the expected size of 301 bp. For quantitative analysis (panel C), the same IgY and PHOX2A immunoprecipitated chromatin as that shown in panel B was amplified by means of SYBR-Green chemistry, quantitatively analysed using the ABI Prism™ 7000 Sequence Detection System (Applied Biosystems, CA) and SDS software, version 1.2.3, and expressed as a percentage of the input DNA ± SEM. D) EMSA analysis of the DNA–protein interactions of PHOX2A with the PHOX2B “ATTA” sites. The gel shift assays were made using oligonucleotide probes corresponding to the ATTA core motifs [4]. The labelled probes were incubated without nuclear extracts (lanes 1, 6, 11, 16, 21) or with nuclear extracts obtained from IMR32 cells (lanes 2–5, 7-10, 12-15, 17-20, 22-25). The competitions were carried out by adding a molar excess of unlabelled wild-type (lanes 3, 8, 13, 18, 23) or mutated oligonucleotides (lanes 4, 9, 14, 19, 24) and the supershift experiments by pre-incubating the nuclear extracts with anti-PHOX2A antibody (lanes 5, 10, 15, 20, 25). The roman numbers I and II on the left indicate the specific retarded complexes obtained using IMR32 nuclear extracts; the arrow indicates the supershifted complexes containing PHOX2A.
