# Supplementary material for: PHOX2A and PHOX2B are differentially regulated during retinoic acid-driven differentiation of SK-N-BE(2)C neuroblastoma cell line
Source: Exp Cell Res. 2016 Mar 1;342(1):62–71. doi: 10.1016/j.yexcr.2016.02.014 (PMC4819706; doi:10.1016/j.yexcr.2016.02.014)
Supplement: Supplementary file 3 — Supplementary material [file mmc3.doc]

**SUPPLEMENTAL MATERIALS AND METHODS**

**Phase-contrast microscopy**

Subconfluent SK-N-BE(2)C cells grown on glass coverslips were fixed with 2% glutaraldehyde in cacodylate buffer, pH 7.4, for 1 h at RT. After three washes in cacodylate buffer, pH 7.4, and a rinse in double distillate H_2_O, the coverslips were mounted on glass slides with Mowiol (Sigma-Aldrich/ Fluka). The samples were observed through a Zeiss Axioplan microscope using a 40x 1.3 Neofluar objective. The phase-contrast images were acquired using a Zeiss Axiocam HRM digital camera.

**Total RNA extraction and Northern blot analysis**

Total RNA was prepared, and the Northern blot analyses were performed as previously described [5]. The RNA was hybridised with human *ASH1* cDNA probes (10^6^ cpm/ml) obtained from SH-SY5Y cells by means of RT-PCR using the upper 5’- CAC CTT TTT TGC TCC CAC TCT AAG-3’ primer and the lower 5'-TCT TTT CCT TTT CTC CCC CTC CCA-3' primer (*HASH1;* GenBank: NM_L08424). A human 18S cDNA probe (nucleotides 715-794; Ambion) was used as a control to check the quality of the RNAs and normalise the previously obtained signals.

**Plasmid construction**

A Nco I-Nco I genomic fragment (-1529/+381) was obtained from one of the six clones isolated from the screening of a commercial human genomic library (described in [5]), and cloned in the proper orientation in the Nco I site of pGL4basic (construct -1.5/pGL4).

The -4.5/pGL4 construct (-4678/+381) was generated by digesting a genomic fragment containing more than 5 Kb of the *PHOX2A* 5’-flanking region with Sac I, and cloned into the -1.5/pGL4 vector digested with Sac I, the sites of which were located in the *PHOX2A* 5’-flanking sequence and in the polylinker of the vector. The -1.2/pGL4 (Pst I-Nco I) vector, which spans the region from -1162 to the first 48 nt of the human *PHOX2A* coding sequence was obtained by digestion with Kpn I-Nco I of the same region cloned in pGL3basic [5]. Digestion of the -1.2/pGL4 with Sac I, blunting, and re-ligation was used to generate the Sac I-Nco I (-1.2/pGL4 Sac I; -71/+381) construct. The -6.6/pGL4b construct was obtained by amplifying the region spanning –6724/-3661 and containing the Kpn I and Sph I sites, using the Expand High Fidelity PCR System (Roche Diagnostics SpA, Monza, Milan, Italy). The Kpn I-Sph I region was amplified using upper primer 5’ – AAC CCA CCT GAG CTT TCT GCC CTG G - 3’ (-6724/-6700), and lower primer: 5’ – CCC CAG TGA AGC TGA CCA GCA CT - 3’ (-3661/-3683). The 3064 bp fragment was sub-cloned in pCR2.1 (Invitrogen Ltd., Paisley, UK) and sequenced on both strands. The -6536/-3700 fragment was then excised by means of digestion with Kpn I and Sph I enzyme, and cloned in the Kpn I and Sph I sites of the -4.5/pGL4 vector, the sites of which were located in the *PHOX2A* 5’-flanking sequence and in the polylinker of the vector. The -10.8/pGL4 construct (-10532/+381) was generated by amplifying the region -10532/-6447. The upper primer was 5’- GGT ACC CTT ACT GTC CGG CCA GGG TG - 3’ (-10532/-10514), to which the Kpn I site (underlined) was added. The lower primer was 5’ – AGC GGC GCT GGC CTC AAA AG – 3’ (-6447/-6465). The obtained 4093 bp fragment was sub-cloned by means of TA cloning into the pCR2.1 vector (Invitrogen Ltd., Paisley, UK), and completely sequenced on both strands. The fragment was then excised by means of Kpn I digestion and cloned in the Kpn I sites of the -6.6/pGL4 construct.
